# Dynorphin participates in interaction between depression and non-erosive reflux disease

**DOI:** 10.1007/s10388-022-00955-0

**Published:** 2022-10-16

**Authors:** Yi Wang, Bingduo Zhou, Shengquan Fang, Shengliang Zhu, Tingting Xu, Makan Dilikumaer, Guanwu Li

**Affiliations:** 1 Department of Gastroenterology, Yueyang Hospital of Integrated Traditional Chinese and Western Medicine, Shanghai University of Traditional Chinese Medicine, Shanghai, China; 2 Department of Radiology, Yueyang Hospital of Integrated Traditional Chinese and Western Medicine, Shanghai University of Traditional Chinese Medicine, Shanghai, China

**Keywords:** Dynorphin, Non-erosive reflux disease, Depression, *N*-methyl-d-aspartate, Hospital Anxiety and Depression Scale, Hypersensitivity

## Abstract

**Background:**

To explore the relationships between anxiety/depression and NERD, we focused on dynorphin (Dyn), an important member of visceral hypersensitivity, and its related pathways.

**Methods:**

Pearson’s correlation analysis on patients with NERD and in vivo experiment on NERD rat model. *Part 1*: Pearson’s correlation analysis among serum levels of Dyn, clinical symptoms and HADS scores of NERD patients were carried on. *Part 2*: Wistar rats were randomly divided into 2 groups: control group and model group. The data of pH value, immobility time, serum Dyn concentration, NMDAR1 and SP expression were, respectively, derived from automatic pH recorder, tail suspension test, enzyme-linked immunosorbent assay, immunohistochemistry and immunofluorescence.

**Results:**

*Part 1*: Pearson’s correlation analysis showed that there was a linear correlation between Clinical Symptom (CS) score and HADS score (HAD-A, HAD-D), and the correlation coefficients were 0.385 and 0.273 respectively; the correlation coefficient between lg (Dyn) and lg (CS score) was *r* = 0.441, *P* = 0.002; the correlation coefficient between lg(Dyn) and lg (HAD-D score) was *r* = 0.447, *P* = 0.002. *Part 2*: The pH value of the lower esophagus in the model group was lower than that in the control group (*P* < 0.01). The tail suspension immobility time of model group was significantly longer than that of control group (*P* < 0.01). The serum Dyn concentration and the expression level of NMDAR1 in spinal cord and SP in lower esophageal mucosa of model group were significantly higher than those of control group (*P* < 0.05).

**Conclusion:**

Increased serum dynorphin level may be a sign of correlation between depression and NERD.

## Introduction

As a subtype of gastroesophageal reflux disease (GERD), non-erosive reflux disease (NERD) refers to the presence of reflux-related symptoms causing discomfort, but no esophageal mucosal damage is found in gastroscopy, also known as symptomatic gastroesophageal reflux disease [1], accounting for 50–70% of GERD [2], up to 78–93% in Asia [3]. A large number of people are suffering from NERD. Although NERD has slight damage to the esophagus, the patients still feel obvious heartburn and acid reflux symptoms, and the quality of life has decreased significantly, which indicates that the key pathogenesis of NERD is not the objective pathological damage caused by reflux stimulation. Weakly acidic reflux and non-acid reflux play important roles in the pathogenesis of NERD [4]. Moreover, weakly acidic reflux was the principal reason of reflux symptom occurrence in patients with PPI-refractory NERD [5–7]. In patients with persistent symptoms on PPI therapy who had an esophageal acid exposure within the physiological range and a positive symptom index (SI) for reflux, a temporal relationship between non-acid reflux and symptoms was observed in the majority (77%) of these patients [8, 9]. Patients with NERD always have less esophageal acid exposure and higher pH value than patients with erosive esophagitis (EE). With the deepening of research, more and more attention has been paid to the role of psychological factors in the occurrence of this disease. According to statistics, about 60% of patients with NERD have anxiety, depression, sleep disorders, autonomic nervous dysfunction, accompanied by decreased quality of life [10, 11]. Previous studies have confirmed that psychological factors may cause visceral hypersensitivity through the brain gut axis [12, 13], leading to mild acid reflux and other low-intensity stimulation that is not perceived by normal people, bringing acid reflux, heartburn, chest pain and other symptoms to patients, just like NERD. Negative emotions play an important role in the pathogenesis of NERD, but the relationship between them and the corresponding molecular biological mechanism have not been clarified.

GERD specialty in our hospital is the key specialty of the 11th and 12th Five-Year Plan of the State Administration of Traditional Chinese Medicine (TCM) of the People’s Republic of China. It is also a clinical advantage specialty of TCM in Shanghai. It has been committed to the clinical diagnosis, treatment and scientific research of NERD. In previous studies, we found that the incidence of anxiety in patients with NERD was 46% (mild 24%, moderate 17%, severe 5%), and the incidence of depression was 55% (mild 28%, moderate 14%, severe 13%). Anxiety increased the risk of NERD by 3.09 times and depression increased the risk of NERD by 1.21 times. At the same time, we noticed that the level of serum dynorphin (Dyn) in NERD patients was higher than that in the general population.

Dyn is a kind of endogenous opioid peptide and an important neurotransmitter in the central nervous system. It is widely distributed in the brain and spinal cord and closely related to visceral sensation. The release of Dyn can activate both *N*-Methyl-d-aspartic acid (NMDA) and bradykinin receptor, directly cause intracellular Ca2 + overload, and generate excessive Nitric Oxide (NO) by NMDA-Ca^2+^—Nitric Oxide Synthase (NOS)/NO pathway, resulting in pain and nerve sensitization [14, 15].

Based on the above research background, we speculate that the increased serum Dyn level may be a marker of the interaction between NERD and negative emotions, and Dyn may participate in the esophageal sensitization process by activating NMDA signaling pathway. To further verify this hypothesis, we have carried out in-depth clinical and animal studies simultaneously. The results are reported as follows.

## Materials and methods

### Clinical study

#### Source of cases and ethical statement

In this study, all the cases meeting the diagnostic criteria and inclusion criteria of NERD, were outpatients of GERD and Digestive Department in Yueyang Hospital of Integrated Traditional Chinese and Western Medicine Affiliated to Shanghai University of Traditional Chinese Medicine from March 2017 to February 2018. The observational study was designed in consistence with guidelines of the Declaration of Helsinki. The study protocol has obtained the approval from the ethics committee of our hospital.

#### Diagnostic criteria of NERD

According to *Evaluation of Gastroesophageal Reflux Disease (2017) *[16]:

1. Clinical manifestations: heartburn and reflux are typical and common symptoms. Other rare or atypical symptoms include one or more of the following: chest pain, epigastric pain, burning sensation in epigastric region, belching, cough, throat discomfort, asthma, tooth erosion, etc.;
2. Endoscopy: no esophageal mucosa damage or Barrett's esophagus (BE) was found.
3. 24 h pH–impedance combined monitoring shows abnormal esophageal acid exposure (Demeester score was greater than 14.72).

#### Inclusion criteria of NERD

1. the diagnostic criteria of NERD;
2. more than 8 of GerdQ score;
3. positive finding in PPI test;
4. clear consciousness and subjective expression of discomfort symptoms;
5. 18–70 years old and regardless of gender.

#### Exclusion criteria of NERD

1. Patients with one of the following diseases: peptic ulcer, history of gastroesophageal or duodenal surgery, gastrinoma, primary esophageal motility disorders (such as achalasia, loss of esophageal peristalsis, distal esophageal spasm, Jackhammer esophagus), upper gastrointestinal malignant lesions, drug-induced esophagitis and psychosis;
2. Pregnant women and lactating women;
3. A researcher directly involved in this observational study.

#### Research methods

According to the linear correlation analysis, the formula of sample size was *n* = 4*(*u*_α_ + *u*_β_)^2^/ln ((1 + *r*)/(1 − *r*))^2^ + 3, *α* = 0.05, *β* = 0.1, *r* = 0.7*,* *n* ≥ 17. To ensure the accuracy of the experimental data and the reliability of the results, the collected blood samples were detected with the same kit and standard. Six standard holes were set in the 96-well plate, and each patient was set with a replicate. Therefore, 45 patients who met the NERD inclusion criteria but not the exclusion criteria were expected to be included in this study.

First, we planned to recruit 180 patients with suspected GERD, who met more than 8 of GerdQ score, positive finding in PPI test, clear consciousness and 18–70 years old. Of course, these subjects might include patients with EE, BE, functional heartburn (FH), reflux hypersensitivity (RH) and other diseases excluding NERD. Second, electronic gastro-duodenoscopy was performed on these patients under off-PPI conditions to exclude patients with EE and BE. Third, patients with suspected NERD completed high-resolution esophageal manometry and 24-h esophageal multichannel intraluminal impedance–pH monitoring as required to find confirmed NERD patients who showed abnormal esophageal acid exposure (Demeester score was greater than 14.72). Esophageal manometry helped us determine the location of the upper edge of lower esophageal sphincter (LES) and exclude primary esophageal motility disorders. The pH electrode of impedance–pH monitoring was located 5 cm above the upper edge of LES. Subsequent observational study included demographic information, Clinical symptom (CS) score, Hospital Anxiety and Depression Scale (HADS) score and serum Dyn level of confirmed patients with NERD.

##### Clinical symptom (CS) score

Based on the diagnostic criteria of *Evaluation of Gastroesophageal Reflux Disease (2017)* [16], the clinical symptom scale is developed according to the severity of symptoms, dividing scores into four grades: asymptomatic, mild, moderate and severe. The asymptomatic means that such symptoms have never occurred; the mild means occasional, less than once a day, not affecting work and rest; the moderate means the frequency of occurrence is between 1 and 3 times a day; the severe means that symptoms occur more than 3 times a day, which affect work and rest. The main symptoms (heartburn, sour regurgitation, retrosternal pain, nausea and belching) are scored as 0, 2, 4 and 6, respectively; secondary symptoms (pharyngeal obstruction, abdominal distension, insomnia, etc.) are scored as 0, 1, 2, and 3, respectively. The higher the score, the more serious the clinical symptoms.

##### Hospital Anxiety and Depression Scale (HADS) score

HADS is mainly used for screening anxiety and depression symptoms. The scale is a self-rating scale with 14 items and consists of 2 subscales, 7 items for anxiety (HADS-A) and 7 items for depression (HADS-D). Each item is scored by Likert 4 grades (0–3 points), and the range of each subscale is 0–21 points. The higher the score, the more serious the anxiety and depression. The total score of 0–7 is normal, the total score of 8–10 is mild depression or anxiety, the total score of 11–14 is moderate depression or anxiety, and the total score of 15–21 is severe depression or anxiety.

##### Enzyme-linked immunosorbent assay (ELISA) used to measure the serum Dyn level

Main reagent was Human Dyn ELISA kit, produced by Shanghai Yuanye Bio-Technology Co., Ltd. Main instruments included high-speed freezing centrifuge 3–18 K (Sigma, Germany), water-proof constant temperature incubator GSP-9160MBE (BoXun Biological Instrument Co., Ltd, Shanghai China) and Microplate Reader Epoch (BioTek, USA).

Operation steps as follows:

1. 5 ml of fasting blood was collected from elbow vein. After standing for 30 min, the blood samples were centrifuged at 3000 *r*/min for 15 min. The upper serum was collected from 45 patients;
2. the 96-hole plate was removed from the aluminum foil bag after 20 min at room temperature;
3. there were 6 standard holes, 90 sample holes (45 samples, each with a double hole), and different concentrations of 50 μL standard solution were added to each standard hole;
4. after 10 μL sample solution was added to the sample holes, 40 μL diluent was added, with blank holes not added;
5. in addition to blank holes, 100 μL HRP-labeled antibody was added to each of standard and sample holes; the reaction holes were sealed with a sealing film and incubated in a 37 ℃ water bath or incubator for 60 min;
6. the liquid of each hole was discarded, patted dry on the absorbent paper and filled with detergent; after placed for 1 min, the detergent of each hole was thrown away; the plate was patted dry on the absorbent paper, and the above steps were repeated for 5 times (the washing machine could also be used to wash the plate);
7. 50 μL substrate A and B were added to all holes, incubating at 37 ℃ for 15 min in dark;
8. 50 μL stop solution was added to each hole; the OD value of each hole was determined at 450 nm within 15 min.

### Animal experiment

#### Animals and ethical statement

Sixteen healthy male Wistar rats, 6 weeks old, weighing 200 ± 20 g, clean grade, were provided by Shanghai SLAC Laboratory Animal Co., Ltd. The temperature of animal feeding room in our hospital was controlled at 20 ± 2 ℃, the humidity was controlled at 50 ± 10%, the light cycle was 12/12 h and the high pressure sterilized bedding material was used. The rats were free to eat standard feed and drink water. The experiment began after a week of adaptive feeding. All animal experiments were conducted strictly in accordance with the guide for the care and use of laboratory animals and approved by the ethics committee of our hospital.

#### Main reagents and instruments

Rat Dyn ELISA Kit, produced by Shanghai Yuanye Bio-Technology Co., Ltd. Microplate Reader Epoch produced by BioTek, USA.

#### Experimental methods

##### Preparation of rat model of non-erosive reflux disease

According to Zayachkivska et al. [17, 18], the rat model of non-erosive reflux disease was established on both fructose intake and psychological stress. Wistar rats were given free fructose water (200 g/L), placed in restraint cages (18*12*13 cm), and immersed vertically to the level of the xiphoid process in a water bath of 22 ± 2 ℃ for 2 h a day for continuous 28 days.

##### Grouping and treatment of experimental animals

Sixteen Wistar rats were randomly divided into two groups: control group and model group, 8 rats in each group. The model group was treated as mentioned above and the control group was not given any treatment.

##### Determination of pH value of the lower esophagus of rats

After deep anesthesia with pentobarbital sodium, the pH electrode of automatic pH recorder was placed 1 cm above the gastroesophageal junction, and the pH value of the lower esophagus was recorded 1 min later.

##### Behavioral observation of rats

The tail suspension test was used to observe the behavior of rats in each group. The tail suspension experiment provides an unavoidable oppressive environment. By recording a series of parameters in the process of the animal’s desperate immobility in this environment, the behavioral despair state can be quantitatively reflected. This behavioral despair model is similar to the depression state. Detailed process [19]: in a quiet environment, the tail of the rat was fixed and suspended with a rubber strip about 3 cm away from the end, and the head of the rat was upside down, about 20 cm away from the bottom. When the rat showed passive suspension and the limb movement disappeared, it was regarded as immobile state. A total of 6 min was observed, and the accumulated immobile time of the rat's tail suspension within final 4 min was recorded.

##### Determination of serum Dyn of rats

After deep anesthesia with pentobarbital sodium, the heart was exposed, the needle was inserted into the left ventricle, and 0.8–1.2 ml blood was extracted. After placed for 30 min, the blood samples were centrifuged at 3000 *r*/min for 15 min. The serum Dyn level was determined by ELISA. The operation steps were the same as operation steps of ELISA used to measure the serum Dyn level above.

##### Immunohistochemistry of spinal cord and esophagus in rats

**Preparation of tissue sections of spinal cord and esophagus** After deep anesthesia with pentobarbital sodium, the thoracic cavity was opened and intubated through the left ventricle to the ascending aorta. 200 ml of 0.9% sodium chloride solution was pressurized and perfused to flush blood of the whole body, with the right atrial appendage cut open, until the liver became completely white. After the colorless flushing solution flowed out from the right atrial appendage, 500 ml of 4% paraformaldehyde fixation solution (pH 7.4) precooled at 4 ℃ was perfused for 1 h until the limbs and spine became hard, and then the spinal cord (T1–T6) and the lower esophagus (15–2 mm above the lower esophageal sphincter) were fixed in paraformaldehyde and embedded in paraffin. According to the anatomic localization of *Histology And Embryology Colour Atlas*, serial coronal and cross-sectional sections (40 μm) were performed. After HE staining, the pathological changes of lower esophageal mucosa were observed under microscope; the expression of NMDAR1 in the posterior horn of spinal cord was observed by Substance P (SP) immunohistochemical staining and the expression of SP in the lower esophageal mucosa was observed by immunofluorescence staining.

**Immunohistochemical SP method** (1) Paraffin sections were made dewax to water; (2) PBST liquid was used to clean each section for 10 min; (3) All sections were soaked in 3% H_2_O_2_ for 15 min, which were protected from light to inactivate endogenous peroxidase; (4) PBST liquid was used to clean each section for 30 min; (5) Citrate was used to repair the antigen for 30 min, temperature of water kept between 92 and  99 ℃; (6) PBST liquid was used to clean each section for 30 min; (7) All sections were soaked in 0.1% Triton X-100 for 10 min to increase the permeability of the membrane; (8) PBST liquid was used to clean each section for 30 min; (9) After drying the slices, we dripped the 10% goat serum on the sections and then incubated them at room temperature for 60 min; (10) All the slices were dried again and were incubated with primary antibody (1/200 in diluent) overnight at 4 ℃; (11) Taken out of the fridge, each piece was first washed in PBST for discontinuation of reaction and then was cleaned in PBST liquid for 30 min; (12) After drying the slices, we dripped the second antibody on the sections and then incubated them at room temperature of 37 ℃ for 30 min; (13) PBST liquid was used to clean each section for 30 min; (14) After drying the slices, we dripped HRP on the sections and then incubated them at room temperature of 37 ℃ for 30 min; (15) PBST liquid was used to clean each section for 30 min; (16) DAB solution was used for coloration protected from light. When they showed brown, we washed the slices in pure water; (17) All the slices were rinsed by water for 10 min; (18) Hematoxylin was used for staining for 10 s; (19) All the slices were rinsed by water for 7–10 min; (20) All the slices were dehydrated, sealed by neutral balsam, and observed under light microscope.

**Immunofluorescence** (1)–(11) were the same as immunohistochemical SP method. (12) After drying the slices, we dripped the IgG-FITC of sheep anti-rabbit and then incubated them at room temperature for 60 min; (13) PBST liquid was used to clean each section for 30 min; (14) After dried, the slices were dripped with DAPI; (15) We used glycerin gelatin for seal and then observed them under fluorescence microscope.

**Positive expression of experimental samples** The positive products of the immunohistochemical SP method which appeared thin yellow fine particles, brown yellow particles and brown yellow coarse granules, were analyzed with image analysis software under optical microscope. One slice was selected to represent each specimen, and the expressions of NMDAR1 in the spinal cord of 5 fields of vision at random were observed under the high magnification microscope (10*40). The average optical density (AOD), meaning IOD/area, of the positive reaction site of each field was calculated. The average value of AOD in 5 fields demonstrated the quantity of antigen. The larger the value was, the more the antigen expressed.

The immunofluorescence positive products were light green, obvious green or bright green with dazzling fluorescence under fluorescence microscopy and were analyzed by image analysis software. We chose one slice for each sample, and randomly observed 5 views for the expression of SP in the esophageal mucosa in lower third of esophagus under the high magnification microscope (10*40). Each integrated optical density (IOD) of the positive reaction site was recorded, and the average value of IOD in the 5 fields was calculated to represent the quantity of antigen expression. The larger the quantity, the stronger the positive expression.

### Statistical analysis

SPSS 21.0 statistical package (provided by IBM, USA) was used for data entry and processing. The data accorded with normal distribution were expressed as mean ± standard deviation (*x* ± *s*), or as median (four quantile range), that is, M (*P*_25_, *P*_75_). When the data accorded with normal distribution and homogeneity of variance, one-way analysis of variance (ANOVA) and LSD-*t* test were used for the intergroup comparisons, or nonparametric test was used. Pearson’s correlation analysis was used for linear correlation analysis. For normality and homogeneity of variance tests, a statistical difference was defined as *P* < 0.1, but for other tests a statistical difference was defined as *P* < 0.05 and a statistically significant difference was defined as *P* < 0.01.

## Results

### General information

During the study period, we had actually recruited 180 patients with suspected GERD, containing 67 patients with EE, 4 patients with BE, and 109 patients with suspected NERD. In patients with suspected NERD, 50 of them showed abnormal esophageal acid exposure as confirmed NERD patients, including 5 cases met the exclusion criteria (2 cases of gastric antral ulcer, 1 case of subtotal gastrectomy, 1 case of distal esophageal spasm and 1 case of Jackhammer esophagus), so finally a total of 45 confirmed cases of NERD (14 males and 31 females) were included and their data were collected. The oldest was 70 years old and the youngest was 24 years old, with a median of 56 years old. The longest course of NERD was 30 years and the shortest was one month, with a median of 42 months. There were 2 cases of chronic hepatitis B, 2 cases of fatty liver, 1 case of gallstone, 7 cases of hypertension, 1 case of lacunar infarction, 1 case of emphysema, 1 case of subacute thyroiditis, 3 cases of hyperlipidemia, 1 case of type 2 diabetes, and 1 case of gout.

### Correlation analysis between CS score and HADS score

Pearson’s correlation analysis showed that there was a linear correlation between CS score and HADS score (HAD-A, HAD-D), and the correlation coefficients were 0.385 and 0.273, respectively (Table 1).

**Table 1:** Correlation analysis between CS score and HADS score

Scale	CS	HADS	
		HAD-A	HAD-D
CS	1	0.385^a^	0.273^a^
HAD-A	0.385^a^	1	0.535^a^
HAD-D	0.273^a^	0.535^a^	1

^a^*P* < 0.01

### Correlation analysis of CS, HADS and serum Dyn level

**Fig. 1:**
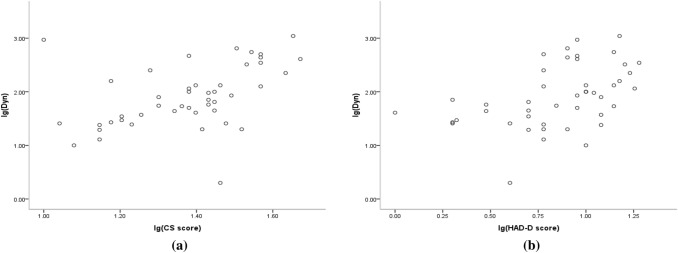
Pearson’s linear correlation analysis. **a** Scatter diagram of lg(Dyn) and lg(CS score). **b** Scatter diagram of lg(Dyn) and lg(HAD-D score)

The data obeyed normal distribution after log transformation X’ = lg(X). Pearson’s correlation analysis showed that the correlation coefficient between lg (Dyn) and lg (CS score) was *r* = 0.441, *P* = 0.002, which was statistically significant. Meanwhile, the correlation coefficient between lg(Dyn) and lg (HAD-D score) was *r* = 0.447, *P* = 0.002. It was implied that there was a linear positive correlation between CS score, HAD-D score and serum Dyn value (Tables 2, 3 and Fig. 1).

**Table 2:** Logarithm conversion of Dyn value

Index	*n*	Raw data (pg/ml)	Lg (Dyn)
		*M* (*P*_25_, *P*_75_)	($\overline{x} \pm s$)
Dyn	45	70.79 (28.21, 237.53)	1.91 ± 0.58

**Table 3:** Correlation analysis of CS score, HAD-D score and serum Dyn value

lg	CS	HADS	
		HAD-A	HAD-D
Dyn	0.441^a^	0.253^b^	0.447^a^

^a^*P* < 0.01

^b^*P* > 0.05

### Comparison of pH value of lower esophagus between two groups

**Fig. 2:**
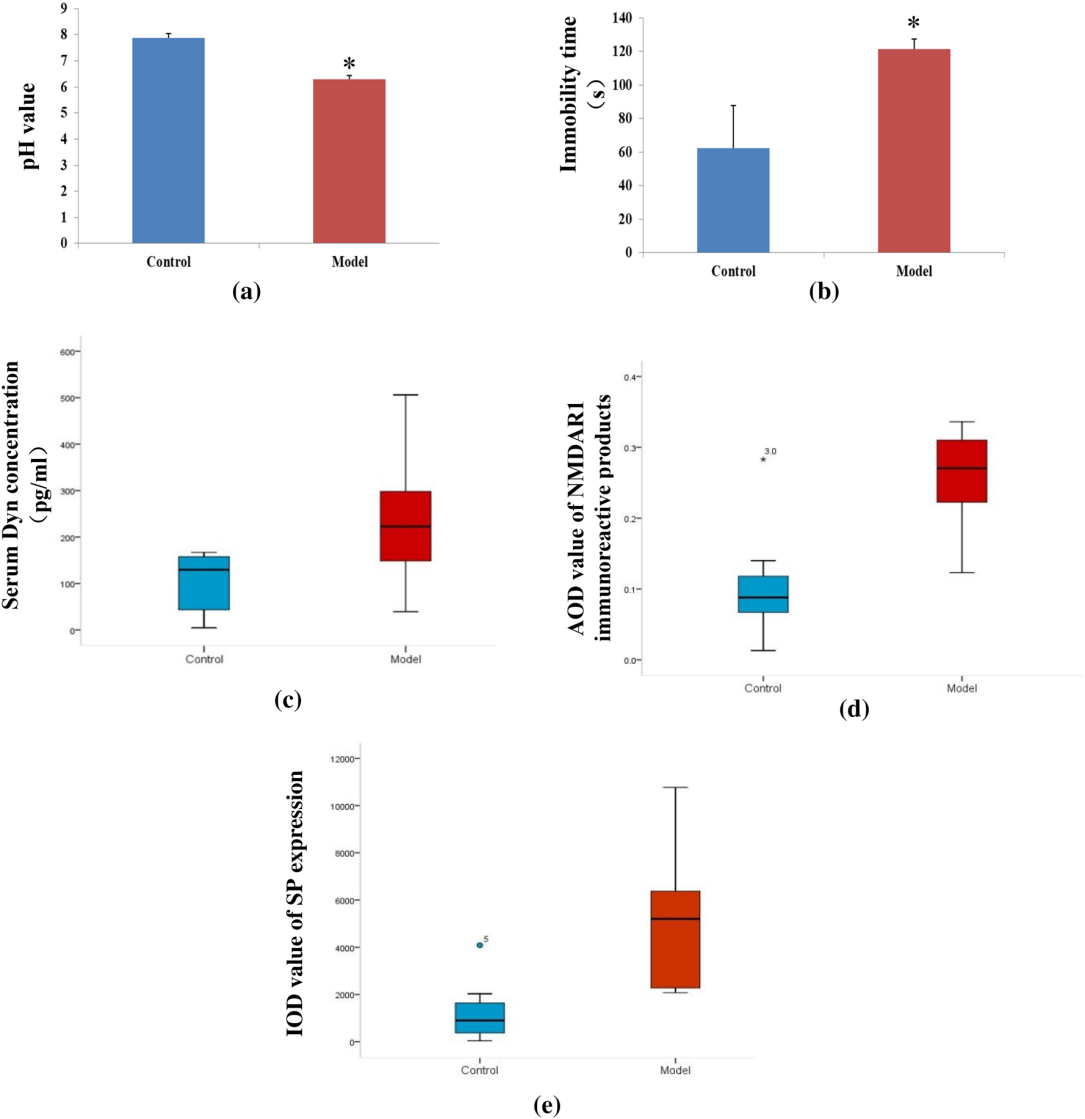
**a**–**e** showed pH value of lower esophagus, immobility time of tail suspension, serum Dyn concentration, AOD value of NMDAR1 immunoreactive products and IOD value of SP immuno-positive products in two groups respectively. The data was presented as mean ± standard deviation (SD) or median (four quantile range), *n* = 8 rats per group. *P < 0.01, Model group vs. Control group. ★ & • represented outliers

The pH value of the lower esophagus in the model group was lower than that in the control group (*P*<0.01), indicating that there was pathological acid reflux in the model group (Table 4 and Fig. 2a).

**Table 4:** pH value of lower esophagus, immobility time of tail suspension, serum Dyn concentration, AOD value of NMDAR1 immunoreactive products in spinal cord, IOD value of SP immuno-positive products in lower esophageal mucosa of rats in two groups

Group	*n*	pH value	Immobility time (s)	Dyn (pg/ml)	AOD of NMDAR1	IOD of SP
Control	8	7.88 ± 0.15	62.25 ± 25.42	129.71 (24.09, 162.32)	0.09 (0.06, 0.13)	334.83 (247.94, 463.71)
Model	8	6.28 ± 0.16△	121.25 ± 6.18*	222.83 (143.84, 321.38)◆	0.27 (0.21,0.31)*	956.50 (596.36, 1909.83)◆

Compared with the control group, ^△^*P* < 0.001, *P < 0.01,^◆^*P* < 0.05

### Comparison of immobility time between two groups

The tail suspension immobility time of model group was significantly longer than that of control group (*P* < 0.01), indicating that rats of model group suffered from depression (Table 4 and Fig. 2b).

### Comparison of serum Dyn concentration between two groups

The serum Dyn concentration in the model group was higher than that in the control group (*P* < 0.05) (Table 4 and Fig 2c).

### Comparison of NMDAR1 expression in spinal cord between two groups

**Fig. 3:**
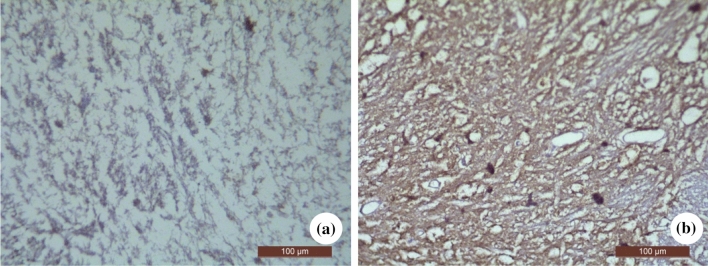
Immunohistochemical staining pictures of expression of NMDAR1 protein in spinal cord of rats in two groups (Original magnification: × 400). **a** Control group; **b** Model group

The expression level of NMDAR1 in spinal cord of model group was significantly higher than that of control group (*P* < 0.01) (Table 4 and Figs. 2d, 3).

### Comparison of SP expression in lower esophageal mucosa between two groups

**Fig. 4:**
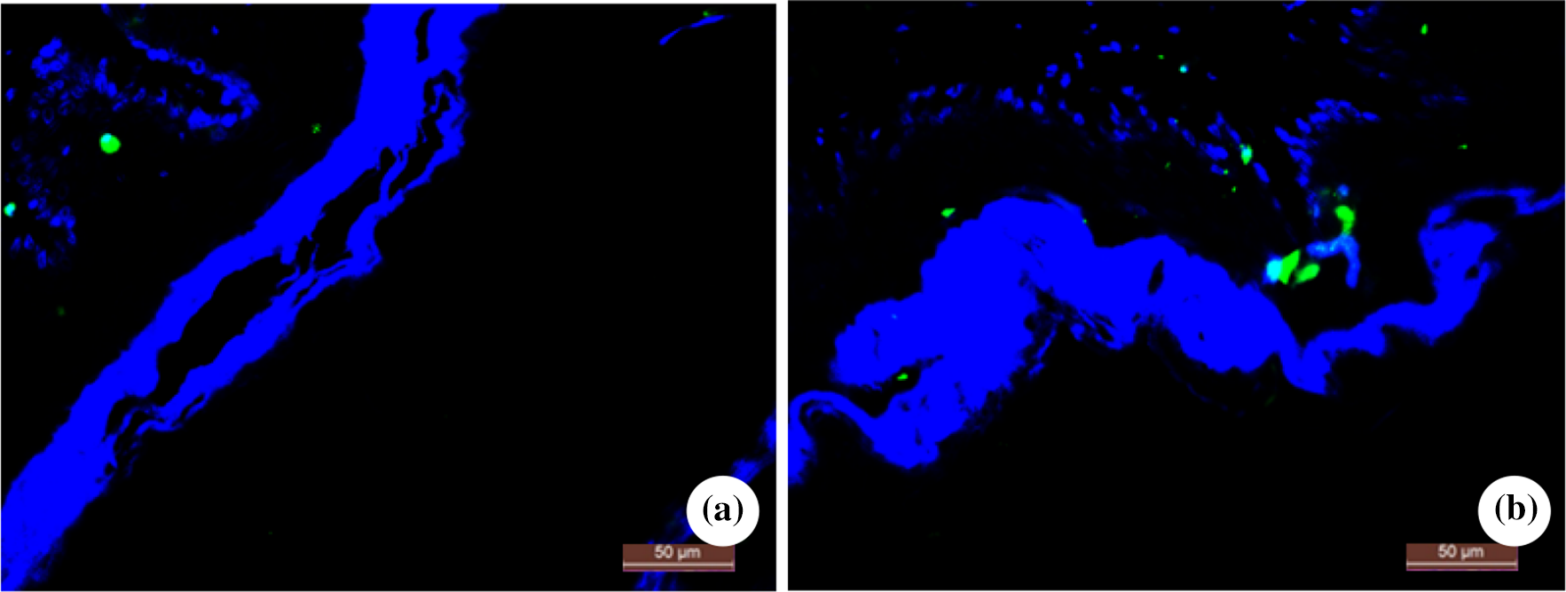
Immunofluorescence staining pictures of expression of SP protein in lower esophageal mucosa of rats in two groups (Original magnification: × 400). **a** Control group; **b** Model group

The expression level of SP in the model group was significantly higher than that in the control group (*P* < 0.01) (Table 4 and Figs. 2e, 4).

## Discussion

Gastroesophageal reflux disease (GERD) is a kind of disease that gastric contents flow back into the esophagus, causing patients’ discomfort and/or complications. The global prevalence rate of GERD is 8–33% [20, 21], and the latest reported prevalence rate in China is 19.89% [22], which is increasing year by year. As a subtype of GERD accounting for more than a half, NERD is currently attracting considerable interest because of its high incidence rate and low response rate to proton pump inhibitor (PPI) [23, 24]. At present, it is considered that the negative psychological state is closely related to the onset of NERD. Chandran et al. [25] found that depression could aggravate the symptoms of reflux, thus reducing the quality of life of patients, and improving the psychological state could help alleviate the problem of reflux. Chen et al. [26] used symptom checklist 90 revised (SCL-90-R) questionnaire to assess psychological disorders of 438 patients with persistent GERD symptoms. The results made it known that the scores of depression, anxiety and paranoia in patients with NERD were significantly higher than those in patients with EE. Oh et al. [27] declared that the main factor affecting the quality of life of NERD patients was the psychological state (anxiety and depression), rather than the severity of symptoms. Avidan [28] revealed that the frequency of heartburn in patients with mental disorders was higher than that in the general population, meaning that mental disorders might make reflux symptoms more easily perceived. Simrén et al. emphasized that in functional gastrointestinal diseases, anxiety and depression could affect the severity of symptoms by regulating visceral sensitivity [13, 29]. For example, visceral hypersensitivity could cause slight expansion, physiological acid reflux and other low threshold stimulation intensity that were not perceived by normal people, resulting in acid reflux, heartburn, chest pain and other symptoms.

Dyn, as a group of neuropeptides related to stress, has strong opioid activity existing in the spinal dorsal horn and brain tissue. In 1988, Spampinato et al. found that intrathecal injection of Dyn (1–32) could not only produce the maximum analgesic effect, but also produce severe hind foot paralysis. Since then, the nociceptive effect caused by Dyn has gradually attracted attention. During inflammatory pain, Dyn biosynthesis increases significantly. It has effect both on analgesia and strengthening pain sensitivity, and the latter often exceeds the former [30]. Ji et al. [31] proved dynorphin A content peaked 3–7 days after nerve injury, and dynorphin A anti-serum intrathecal injection decreased IL-β and TNF-α content a week after nerve injury. The most likely reason is that Dyn participates in the body's pain response and plays a certain protective role in the early stage, but gradually becomes a damaging factor after excessive accumulation. In the pain model caused by chronic inflammation, Dyn in the spinal dorsal horn had pain promoting and neuro-sensitization effects [32]. In Campillo’s study, the nociceptive response threshold decreased significantly and the expression level of Dyn increased in mice anesthetized with remifentanil [33]. Negrete et al. [34] confirmed the endogenous dynorphin/kappa opioid receptor (KOR) system took part in the mechanical allodynia. Furthermore, knockout of the dynorphin gene prevented development of chronic pain in mice. The use of anti-dynorphin A anti-serum could reduce thermal hyperalgesia [35], and the application of anti-dynorphin A anti-serum in the nerve sheath could reduce the abnormal pain after spinal cord trauma [36]. All those have shown the correlation between Dyn and hypersensitivity.

This study has found that there was a linear correlation among serum Dyn value, clinical symptom score and HAD-D score, which were positive correlation, but there was no linear correlation between serum Dyn value and HAD-A score, showing that serum Dyn level may be a sign of the correlation between NERD and depression rather than anxiety. The reason for this result is that the pathogenesis of anxiety is not the same as depression. The former is mainly related to the decrease of GABA, serotonin and norepinephrine; the latter is related to the decline of the functional activities of neurotransmitters, such as serotonin and norepinephrine, the increase of neuroendocrine factors like cortisol and the change of brain structure like abnormal gray matter volume. Many studies have confirmed that depression is relevant to dynorphin [37–39], but few studies supported the relationship between anxiety and dynorphin and some researchers even found genetic inactivation of κ Opioid Receptor (KOR)-Dynorphin signaling resulted in increased anxiety-like behavior [40]. Anxiety may also act on NERD through other opioid-related peptide like nociceptin/orphanin FQ (N/OFQ) rather than dynorphin [41].

While Dyn synthesis increased in the dorsal horn of spinal cord, local tissue inflammation and hyperalgesia increased, indicating that the transmission of continuous nociceptive information at the spinal cord level was not a passive process but an active process of central axis plasticity [42]. Further studies have found that a large amount of Dyn directly or indirectly produces some inflammatory mediators or cytokines through NMDA receptors, so as to strengthen the formation of neuropathic pain. This effect works through spinal NMDA receptors rather than opioid receptors [43, 44] because it can be reversed by NMDA receptor blocker MK-801 [45], leading to the decrease of release of excitatory amino acids and nociceptive response in the spinal cord caused by Dyn [46]. In the periphery, when the gastrointestinal tract is subjected to noxious stimulation, a large amount of SP can be secreted in the gastrointestinal mucosa and enteric nerve in subcutaneous muscularis to participate in the transmission of noxious information and cause pain. SP is an excitatory transmitter released from nociceptive afferent terminals and acts on NK1 receptor in tachykinin receptor family. SP takes part in the sensory transduction of glutamate (Glu), and NMDA receptor and NK1 receptor play a synergistic role in mediating the transmission of spinal pain information [47]. In central sites, such as arcuate nucleus and median eminence of rats, Glu can promote the release of SP through NMDA receptor [48]. Except the relationship of visceral sensory conduction, NMDA and SP are also involved in the occurrence of stress-induced depression. Its internal mechanism is that chronic stress causes the increase of Glu level, over activates NMDA receptor, promotes the expression of SP, and then activates NK1 receptor to induce depressive behavior [49]. This study found that the serum Dyn concentration, NMDAR1 protein in spinal cord and SP expression in lower esophageal mucosa in the model group of NERD with depression were significantly higher than those in the blank control group (*P* < 0.05). Associated with the interaction of the three in the formation of visceral hypersensitivity, we speculate that depression may promote the formation of visceral hypersensitivity by the increase in the expression of Dyn in the posterior horn of the spinal cord, and then participate in the occurrence of NERD; the increase of serum Dyn level is not only an indirect evidence of high expression of Dyn in spinal cord, but also a sign of the correlation between NERD and depression. After Dyn releases from spinal cord, it can directly activate NMDAR and cause a large amount of SP release from peripheral afferent synapses, enhancing visceral hypersensitivity. Therefore, NMDA signal pathway may be the formation mechanism of visceral hypersensitivity in NERD patients with depression.

However, our study has some limitations. First, we designed this study to explore whether Dyn and related pathways were involved in the interaction between NERD and negative emotions like depression and anxiety. Nevertheless, it was hard to obtain the patients’ spinal cord biopsy specimens. There was a risk of bleeding in esophageal biopsy, which was generally carried out on patients with Barrett's esophagus. Therefore, in this study, the serum of patients and spinal cord and esophageal mucosa of rats were taken for preliminary study. Second, based on the results of our study, we speculated Dyn regulated the esophageal sensitization process of NERD with depression by the expressions of NMDAR1 in spinal cord and SP in lower esophageal mucosa from the central and peripheral levels respectively. Although the results of this study showed a relationship between Dyn and depression, the relationship between Dyn and NERD has not been directly demonstrated. In-depth research should include behavioral evaluation of esophageal hypersensitivity on patients and rat models, inflammatory factors related to SP and downstream signaling molecules of NMDA such as cAMP/PKA to directly prove the relationship between Dyn and NERD. Third, according to *Diagnostic criteria* and *Inclusion criteria of NERD* in this study, patients whose esophageal intraluminal impedance–pH monitoring showed abnormal esophageal acid exposure (Demeester score was greater than 14.72) would be recruited in. This caused NERD patients with belching disorders to be included in the study, but patients with belching only would be excluded. It is well known that belching disorders are always related to psychological distress. Although the proportion of patients with complications was less than 20%, it might affect the results of our research to some extent.

In future, more in-depth clinical trials and animal experimental researches should be carried out around the mechanism of interaction between depression and NERD, to find more scientific evidence to clarify the molecular biological mechanism of Dyn and NMDA signaling pathways in spinal cord and brain center involved in the pathogenesis of NERD. It is a very meaningful work not only to the objective assessment of the psychological state of NERD patients but also to the exploration of new targets for treatment.

## Conclusion

Although there existed some limitations of our research, it provided an evidence that increased serum dynorphin level as a sign of correlation between depression and non-erosive reflux disease and NMDA signaling pathway in spinal cord might participate in the formation of esophageal hypersensitivity in patients with NERD, resulting in the interaction between symptoms and depression.

## References

[1] Hershcovici T, Fass R (2010). Nonerosive Reflux Disease (NERD)—an update. J Neurogastroenterol Motil.

[2] Dore MP, Pes GM, Bassotti G, Farina MA, Marras G, Graham DY (2016). Risk factors for erosive and non-erosive gastroesophageal reflux disease and Barrett's esophagus in Nothern Sardinia. Scand J Gastroenterol.

[3] Fock KM, Talley N, Goh KL, Sugano K, Katelaris P, Holtmann G (2016). Asia-Pacific consensus on the management of gastro-oesophageal reflux disease: an update focusing on refractory reflux disease and Barrett's oesophagus. Gut.

[4] Bredenoord AJ (2012). Mechanisms of reflux perception in gastroesophageal reflux disease: a review. Am J Gastroenterol.

[5] Abe Y, Koike T, Saito M (2019). Influence of the pH value of refluxate and proximal extent on heartburn perception in patients with proton pump inhibitor-refractory non-erosive reflux disease. Digestion.

[6] Frazzoni M, Conigliaro R, Melotti G (2011). Weakly acidic refluxes have a major role in the pathogenesis of proton pump inhibitor-resistant reflux oesophagitis. Aliment Pharmacol Ther.

[7] Tsoukali E, Sifrim D (2010). The role of weakly acid-ic reflux in proton pump inhibitor failure, has dust settled?. J Neurogastroenterol Motil.

[8] Viazis N, Keyoglou A, Kanellopoulos AK (2012). Selective serotonin reuptake inhibitors for the treatment of hypersensitive esophagus: A placebo controlled study using esophageal pH-impedancemonitoring. Am J Gastroenterol.

[9] Karamanolis GP, Tutuian R (2013). Role of non-acid reflux in patients with non-erosive reflux disease. Ann Gastroenterol.

[10] Lindam A, Ness-Jensen E, Jansson C, Nordenstedt H, Åkerstedt T, Hveem K (2016). Gastroesophageal reflux and sleep disturbances: a bidirectional association in a population-based cohort study, The HUNT Study. Sleep.

[11] Oparin AA, Balaklytska IO, Morozova OG, Oparin AG, Khomenko LO. Mechanisms of insomnia formation with gastroesophageal reflux disease, taking into account the psychosomatic status in young people.*Wiad Lek.* 2020;73:1365–9. 32759421

[12] Bravo JA, Dinan TG, Cryan JF (2011). Alterations in the central CRF system of two different rat models of comorbid depression and functional gastrointestinal disorders. Int J Neuropsychopharmacol.

[13] Midenfjord I, Polster A, Sjövall H, Törnblom H, Simrén M (2019). Anxiety and depression in irritable bowel syndrome: exploring the interaction with other symptoms and pathophysiology using multivariate analyses. Neurogastroenterol Motil.

[14] Altier C, Zamponi GW (2006). Opioid, cheating on its receptors, exacerbates pain. Nat Neurosci.

[15] Cheng HY, Penninger JM (2004). DREAMing about arthritic pain. Ann Rheum Dis.

[16] Fisichella PM, Andolfi C, Orthopoulos G (2017). Evaluation of gastroesophageal reflux disease. World J Surg.

[17] Zayachkivska O, Havryluk O, Hrycevych N, Bula N, Grushka O, Wallace JL (2014). Cytoprotective effects of hydrogen sulfide in novel rat models of non-erosive esophagitis. PLoS ONE.

[18] Mizoguchi K, Yuzurihara M, Ishige A, Sasaki H, Chui DH, Tabira T (2000). Chronic stress induces impairment of spatial working memory because of prefrontal dopaminergic dysfunction. J Neurosci.

[19] Shinde V, Yegnanarayan R, Shah P, Gupta A, Pophale P (2015). Antidepressant-like activity of flunarizine in modified tail suspension test in rats. N Am J Med Sci.

[20] Gyawali CP, Kahrilas PJ, Savarino E, Tack J, Bredenoord AJ, Pandolfino J (2018). Modern diagnosis of GERD: the lyon consensus. Gut.

[21] El-Serag HB, Sweet S, Winchester CC, Dent J (2014). Update on the epidemiology of gastro-oesophageal reflux disease: a systematic review. Gut.

[22] Gong Y, Zeng Q, Yan Y, Han C, Zheng Y (2019). Association between lifestyle and gastroesophageal reflux disease questionnaire scores: a cross-sectional study of 37442 Chinese adults. Gastroenterol Res Pract.

[23] Dean BB, Gano AD, Knight K, Ofman JJ, Fass R (2004). Effectiveness of proton pump inhibitors in nonerosive reflux disease. Clin Gastroenterol Hepatol.

[24] Park JM, Chi KC (2018). Antireflux surgery is equally beneficial in nonerosive and erosive gastroesophageal reflux disease. Ann Surg Treat Res.

[25] Chandran S, Raman R, Kishor M, Nandeesh HP (2019). The effectiveness of mindfulness meditation in relief of symptoms of depression and quality of life in patients with gastroesophageal reflux disease. Indian J Gastroenterol.

[26] Chen X, Li P, Wang F, Ji G, Miao L, You S (2017). Psychological results of 438 patients with persisting gastroesophageal reflux disease symptoms by symptom checklist 90-revised questionnaire. Euroasian J Hepatogastroenterol.

[27] Oh JH, Kim TS, Choi MG, Lee H, Jeon EJ, Choi SW (2009). Relationship between psychological factors and quality of life in subtypes of gastroesophageal reflux disease. Gut Liver.

[28] Avidan B, Sonnenberg A, Giblovich H (2001). Reflux symptoms are associated with psychiatric disease. Aliment Pharmacol Ther.

[29] Simrén M, Törnblom H, Palsson OS, van Tilburg MAL, Van Oudenhove L, Tack J (2018). Visceral hypersensitivity is associated with GI symptom severity in functional GI disorders: consistent findings from five different patient cohorts. Gut.

[30] Podvin S, Yaksh T, Hook V (2016). The emerging role of spinal dynorphin in chronic pain: a therapeutic perspective. Annu Rev Pharmacol Toxicol.

[31] Ji L, Chen Y, Wei H, Feng H, Chang R, Yu D (2019). Activation of alpha7 acetylcholine receptors reduces neuropathic pain by decreasing dynorphin A release from microglia. Brain Res.

[32] Malan TP, Ossipov MH, Gardell LR, Ibrahim M, Bian D, Lai J (2000). Extraterritorial neuropathic pain correlates with multisegmental elevation of spinal dynorphin in nerve-injured rats. Pain.

[33] Weinbroum AA (2017). Postoperative hyperalgesia—a clinically applicable narrative review. Pharmacol Res.

[34] Negrete R, García Gutiérrez MS, Manzanares J, Maldonado R (2017). Involvement of the dynorphin/KOR system on the nociceptive, emotional and cognitive manifestations of joint pain in mice. Neuropharmacology.

[35] Luiz AP, Schroeder SD, Rae GA, Calixto JB, Chichorro JG (2015). Contribution and interaction of kinin receptors and dynorphin A in a model of trigeminal neuropathic pain in mice. Neuroscience.

[36] Lough C, Young T, Parker R, Wittenauer S, Vincler M (2007). Increased spinal dynorphin contributes to chronic nicotine-induced mechanical hypersensitivity in the rat. Neurosci Lett.

[37] Zan GY, Sun X, Wang YJ (2022). Amygdala dynorphin/κ opioid receptor system modulates depressive-like behavior in mice following chronic social defeat stress. Acta Pharmacol Sin.

[38] Al-Hakeim HK, Zeki Al-Fadhel S, Al-Dujaili AH (2020). In major depression, increased kappa and mu opioid receptor levels are associated with immune activation. Acta Neuropsychiatr.

[39] Newton SS, Thome J, Wallace TL (2002). Inhibition of cAMP response element-binding protein or dynorphin in the nucleus accumbens produces an antidepressant-like effect. J Neurosci.

[40] Baird MA, Hsu TY, Wang R (2021). κ opioid receptor-dynorphin signaling in the central amygdala regulates conditioned threat discrimination and anxiety. eNeuro.

[41] Fulford AJ (2015). Endogenous nociceptin system involvement in stress responses and anxiety behavior. Vitam Horm.

[42] Sapio MR, Iadarola MJ, Loydpierson AJ, Kim JJ, Thierry-Mieg D, Thierry-Mieg J (2020). Dynorphin and enkephalin opioid peptides and transcripts in spinal cord and dorsal root ganglion during peripheral inflammatory hyperalgesia and allodynia. J Pain.

[43] Woods AS, Kaminski R, Oz M, Wang Y, Hauser K, Goody R (2006). Decoy peptides that bind dynorphin noncovalently prevent NMDA receptor-mediated neurotoxicity. J Proteome Res.

[44] Hall SM, Lee YS, Hruby VJ (2016). Dynorphin A analogs for the treatment of chronic neuropathic pain. Future Med Chem.

[45] Hillhouse TM, Negus SS (2016). Effects of the noncompetitive N-methyl-d-aspartate receptor antagonists ketamine and MK-801 on pain-stimulated and pain-depressed behaviour in rats. Eur J Pain.

[46] Chen Y, Xiang L, Liu J, Zhou D, Yu H, Wang Q (2012). A non-opioid pathway for dynorphin-caused spinal cord injury in rats. Neural Regen Res.

[47] Chen W, Zhang G, Marvizón JC (2010). NMDA receptors in primary afferents require phosphorylation by Src family kinases to induce substance P release in the rat spinal cord. Neuroscience.

[48] Caruso C, Durand D, Watanobe H, Lasaga M (2006). NMDA and group I metabotropic glutamate receptors activation modulates substance P release from the arcuate nucleus and median eminence. Neurosci Lett.

[49] Zhou Q, Grevés PL, Ragnar F, Nyberg F (2000). Intracerebroventricular injection of the N-terminal substance P fragment SP(1–7) regulates the expression of the N-methyl-D-aspartate receptor NR1, NR2A and NR2B subunit mRNAs in the rat brain. Neurosci Lett.

